# Practical Observations on the Preservation of Health and the Prevention of Diseases: Comprising the Author's Experience on the Disorders of Childhood and Old Age, on Scrofula, and on the Efficacy of Cathartic Medicines

**Published:** 1838-07

**Authors:** 


### Art. XV.

*Practical Observations on the Preservation of Health, and the Prevention of Diseases: comprising the Author's Experience on the Disorders of Childhood and Old Age, on Scrofula, and on the Efficacy of Cathartic Medicines.* By Sir Anthony Carlisle, f.r.s. President of the Royal College of Surgeons, and Surgeon to the Westminster Hospital.—*London,* 1838. 8vo. pp. xlvii. 154.

It has pleased Sir Anthony Carlisle to present once more to the public some old observations, united with some new ones, in a handsome volume, much too dear, although embellished, like his former publication on Old Age, with a title-page in red letters and black. The work is dedicated, out of pure respect for senility, to the Dowager Lady Cork; and it is addressed, the author says, "to a class of readers unknown in preceding ages—to well-educated persons, who have discovered the deficiencies of classical learning in physic, and who begin to doubt the mysteries involved in dead languages." He apprehends that, in publishing this volume, he is sacrificing worldly professional interests to a sense of public duty. To guard, moreover, the unthinking from any suspicion of his want of "profundity" in consequence of this gay appearance in a popular work, he takes care to refer them to two out of his numerous former productions, the alleged Discovery of the Uses of the Spleen and Thyroid Gland, and the Physiological Observations on Glandular Structures; and to obviate a suspicion, which might arise, he conjectures, from the absence of quotations, that the author is unlearned, he declares that he "possesses and employs the established wisdom of preceding authors." It is not for us to be captious with so ardent a cultivator of the medical art; and therefore, although Sir Anthony is no longer young, we accept the red letters, the dedication to the elderly lady, and the profession of extra-profundity and all manner of learning, with the utmost possible good humour. We may regret to see how strongly, withal, Sir Anthony, is prepossessed against the double-columns of the classics of physic; and doubt his assertion that "the most skilful practitioners in physic have been generally trained in the schools of surgery;" but come in what shape they may, the remarks of a veteran observer, trained up under the eye of Hunter, and full of experience gathered in the great field of London practice, must have something valuable in them, and should be received with respect.

We shall therefore merely avow the surprise with which we have read forty-seven pages of Introductory Exposition, in which certain ideas are repeated about forty-seven times; the ideas being the very novel ones, that medicine and surgery are becoming less conjectural as they become improved, and that ignorant practitioners are rash, and unprincipled practitioners presuming, and experienced men cautious; and the conclusions being, that the best books are those "written by experienced men in advanced age," of whom the author says that he is one, and one who has "contributed lavishly" to render the profession respectable, to augment medical science, and to improve surgical apparatus.

After an interesting account of the topography of London, Sir Anthony Carlisle makes some useful, although not very new observations on its climate and atmosphere. The London air, when dry, is loaded with the dust of ashes and soot, and horse-dung, and gravel and granite. Dr. Watson (Bp. of Llandaff) found, if we remember correctly, that chopped hay formed a material ingredient. The result of this "chaos of eternal smoke" and dust, is some difficulty of breathing and cough, but chiefly in those newly arrived from the country. But there is also "a volatile corruption" of which the effects seem to be fevers which often attack the visitors to the metropolis. We believe that no town affords more instructive instances both of the immunity from and prevalence of malignant fevers; immunity from them in the wider streets and squares, and in the better ventilated houses, among the better nourished inhabitants; and their prevalence in courts and allies, and

> "in many a sullen bay
> That never felt the freshness of the breeze,"

where every cause of deteriorated health is accumulated. Within the prisons of the metropolis, also, it would seem, from the testimony of a patient of Sir Anthony Carlisle's, whose testimony was worth regarding, the mental apathy and recklessness which form so shocking a part of the scene those receptacles of misery display, are in a great measure the product of confinement in bad air. Yet the general freedom of London from all common nuisances, from bad smells and offensive sights, is one of the wonders of well-ordered communities; but a wonder effected by systematic attention and mechanical arrangements. Nor is it a circumstance indifferent to humane persons, that those whose lot in life it is almost to live in the vast drains and sewers of this city of nearly two millions, without which London would soon be the city of the dead, enjoy good health, soon recover from the accidental wounds or injuries they receive, and appear to suffer as little inconvenience as any other class of labourers. When all is done, however, that medical police can do, it is yet perhaps true that the human being, in these populous cities pent, gradually degenerates; that his offspring, as Sir Anthony Carlisle observes, become more and more feeble, and families become extinguished "through a progressive degeneracy." (p. 23.)

> "I believe," he observes in another page, "that no persons, town-bred in both the male and female lines, ever extend their children to the fourth generation; for they progressively dwindle, and lose the respective sexual characters until their procreative ability ceases." (p. 34.)

Four times the number of children perish, in the London air, of those who die when nursed in the air of the country. He goes on to express his belief that our great manufacturing towns give rise to "a degenerate, enfeebled, and demoralized race of town-bred citizens, who lack that happy union of a sound mind united to a sound body, which was held to be superlatively valuable by Roman statesmen." (p. 27.) There is some truth, perhaps with a small admixture of extravagance, in the following passages; touching upon different points of political economy:

> "If ever men," he says, "should become so far civilized as to cooperate faithfully for each other's good, a further advancement in knowledge might show them the justice of moving whole families from the progressive injury of city residences, from unwholesome localities, and even from certain climates to others more genial, calculated to remove the bad effects of permanent settlements; but these views are Utopian, and in utter opposition to those prevailing doctrines which, I fear, are undermining the bodily constitution of a large portion of our countrymen, and, by sanctioning the unsocial vices of greediness and monopoly, are rapidly driving all Europe into a state of violence and confusion, which may ultimately stop the progress of every science, and (were it not for the indestructible extension of printed books) bring darkness and barbarism again over the civilized world.' (p. 27.)

"Without pretending to be a competent judge of the modern doctrines of political economy, I cannot agree to the cold-blooded restraints and punishments attempted to be fixed upon those who have the strongest animal passions, and to prohibit the most powerful of our race from sinking into selfishness, instead of being the proud and responsible parents of future generations." (p. 35.)

"Whatever the ultimate political or moral results of the practical working of the abstract doctrines for accumulating wealth, and for reducing population may be, it is obvious to a medical philosopher, that the moral and physical debasement of the English character must be the certain consequences; and however flattering the illusory dreamings of these selfish economists may be for a time, the reverie must terminate either in the extermination of a manly, virtuous, and industrious people, or in some dreadful insurrection." (p. 36.)

We discern the observation of the experienced metropolitan practitioner in Sir Anthony's graphic medical sketches of the different classes which compose London society: these will well repay the reader's perusal. The sketches are slight indeed, but expressive, and comprehend many of the causes of the diseases which assail the rich, strike the luxurious, or devastate the poor.

The remarks on diet and clothing are judicious, and such as few medical authorities would now be found to dissent from. Sir Anthony objects to giving much vegetable food, or even much bread, to young children; and reprobates the cusom of exposing town-bred children, especially, to the inconveniences of insufficient clothing. He considers calico preferable to linen, both for personal covering and for beds; and thinks a wash-leather waistcoat worn over a calico shirt is a better defence against cold and damp than flannel. He justly condemns the flimsy materials of ladies' shoes.

Scrofula, Sir Anthony maintains, is not hereditary; yet, as he allows that "this over-delicacy of the frame, which affords the aptitude to scrofulous disease, may be hereditary," we see nothing novel in his arguments on this point, except a more than usually anxious desire to gratify the self-love of parents, who are well satisfied in such cases if their doctors can only keep the word of promise to the ear, and assure them that the malady which disfigures or destroys their children is incidental, and not constitutional. Indeed, the whole chapter on scrofula is the most striking exemplification we have seen for a long time of that species of composition, not unfamiliar to reviewers, where what is true is not new, and what is new is not true. Of the truths we need not speak; of the novelties we can only find room for one or two small specimens.

*Consumption.* "The chest is narrow, and that is vulgarly and erroneously supposed to confine the lungs, and thereby to occasion consumption; which is a scrofulous malady dependent on repeated attacks of feeble inflammation, progressively spoiling the natural texture of the lungs, and, if not prevented, leading to an accumulation of incurably diseased structure." (p. 56.)

*Bronchocele.* "If it should be asked, what can have happened to produce that unnatural enlargement, I reply, that it is a mere dilatation of a congeries of blood-vessels, called the thyroid gland; and that it arises from long-continued exposure of the bare throat to a piercing, damp, and cold atmosphere, which physically impedes the flow of blood through vessels benumbed by a low temperature: similar effects follow chilling exposure of the lips, hands, and feet of feeble and delicate children, who are unjustly condemned to the opprobrium of scrofula." (p. 59.)

In the foregoing statement we have specimens of the author's anatomy and pathology; we will conclude with a still more luminous example of his physiology: but we would ask, first, in reference to the above, how happens it that bronchocele is so frequent in many countries between the tropics, in Asia, Africa, and America; and so rare in the arctic regions? or, to come nearer home, why is it unknown in Caithness and Sutherland, and so rife in the warm and blooming vallies of Hampshire and Sussex?

*General Physiology.* "Throughout animal nature, the circulation of red blood and nervous sensations keep pace with temperature; a general coincidence most remarkable in cold-blooded creatures, such as frogs and fishes, whose powers of motion, like those of our fingers, are benumbed by cold." (p. 60.)

We leave it to Dr. Edwards, Dr. Sharpey, Mr. Owen, and other cultivators of the higher physiology, to profit by and expound the wisdom wrapt up by the President of the Royal College of Surgeons in this dictum: we confess that it is above our mark.

In the same chapter, Sir Anthony confidently asserts that pulmonary consumption may *always* be prevented "by undeviating attentions to clothing, to local residence, to domestic accommodation, and to diet," (p. 63;) and he states that he has "often witnessed recoveries from some of the most direful ravages of scrofulous ulcerations penetrating the spine, the joints of the knees, ankles, elbows, and wrists, under all the disadvantages of London air, and confinement to the same room for more than two years, by the simple and rational adaptations of warmth, clothing, and diet, with very little medicine." (p. 69.) We may admit the fact in the latter part of the sentence; although we deny the truth of the opinion in the former. At the same time we are most ready to allow that the "attentions" referred to, can do a great deal more than is commonly believed, in such cases.

The section on the Preservation of the Health of Children is not very correctly designated, for it rather relates to the preservation of health in general. It is somewhat superficial, but characterized by good sense. Sir Anthony appears to set little value on many of the popular works published within the last few years on this subject; and is evidently little aware of the great quantity of useful information which some of them contain.

Twenty years having passed away since the publication of the first edition of the treatise on the Disorders of Old Age, it comes before many readers almost like a new work; and in this point of view its reputation will not be increased by its reappearance. The more experienced author is still the same enemy to morbid anatomy, and to the general attempts of his professional brethren to advance pathological knowledge that he always was. He speaks of their labours in a tone of vague depreciation, as mystical as absurd, and yet strenuously maintains that the science they cultivate is becoming exact, and ought to be protected by public opinion from the invasion of quacks and pretenders. With this strain every treatise in the book begins; with this strain every treatise ends. We can imagine the morbid anatomists turning round upon him on reading the very first sentence of his republished essay, and pronouncing it at once to be artificial and incorrect.

"When the age of maturity has passed, and the lungs have escaped a derangement of structure most incident to youth, the common dangers to life are to be discovered in disorders of the head, the stomach, the bowels, the blood-vessels, and the liver; and they display themselves by apoplexy, palsy, indigestion, obstructions, inflammations, jaundice, or dropsy." (p. 89.)

Every morbid anatomist, and every practitioner *less* experienced than Sir Anthony, will be inclined to add a few affections to this catalogue, in which the thoracic organs are primarily, seriously, peculiarly, and often fatally affected in old persons. Not a syllable is said, here or elsewhere, of the great liability of old persons to organic changes in the heart and great vessels; facts which morbid anatomy has pretty well established. Such alterations are, perhaps, disregarded by Sir Anthony; for, among the very few new paragraphs in this edition, we find the following:

"Diseases, constituted by alterations of organic structure, are seldom within the reach of physic, unless they invade parts of little consequence to life, when the administrations of surgery may prove useful. Too much attention has been empirically attracted towards anatomy, which is only one of the rudiments of medicine, and common to all well-educated practitioners. But the incurable ravages of disorganized parts afford few indications as to remedies, while they expose the most hopeless and lamentable obstacles to our art." (p. 93.)

To a want of regard for the progressive state of opinions and facts in medicine, similar to that manifested in these remarks, we can alone ascribe the assertion made at p. 126, that "a popular hypothesis is now very prevalent, which attributes nearly all diseases to a disturbed state of the liver," &c. &c. Sir Anthony said this, and truly enough, some twenty years ago; but, if he had considered the vascular, nervous, gastro-intestinal, and humoral theories, not to speak of others, which have occupied the attention of most other medical men in the mean time, he would scarcely have repeated the assertion in 1838.

Among the few practical observations now added we find the following:

"There is an affection, not uncommon to old persons, which resembles apoplexy, arising from scantiness, and perhaps also from poverty, of blood. This is accompanied by faintings, the person having a slow feeble pulse; the faintings happen in the upright position, and after long standing up. Such persons have their lives prolonged by full diet, and the fit is removed by resorting to the horizontal position *instanter*. I think that this condition is often dependent on the habitual stimulus of wine, which, I believe, *thins* the blood, and fails to supply a permanent suitable volume of the circulating medium. Milk diet, and frequent supplies of animal broths, have, under my experience, forced life on for some years beyond the apprehended close." (p. 130.)

The observations on Cathartic Medicines contain nothing that particularly demands our attention; and, upon the whole, we cannot but observe, that the collection of the different treatises comprehended in this showy volume would have been more favorably looked upon, without question, if placed within reach of medical readers at about half the price. To men of moderate reading and experience nothing new is presented; and to the student, such luxuries of medical literature are necessarily postponed. The didactic method, too, has lost its charm; and the bulk of mankind, breathing an atmosphere of general knowledge, are little affected by the most pompous announcements.

